# Exercise-Based Training Strategies to Reduce the Incidence or Mitigate the Risk Factors of Anterior Cruciate Ligament Injury in Adult Football (Soccer) Players: A Systematic Review

**DOI:** 10.3390/ijerph182413351

**Published:** 2021-12-18

**Authors:** Jesús Olivares-Jabalera, Alberto Fílter-Ruger, Thomas Dos’Santos, Jose Afonso, Francesco Della Villa, Jaime Morente-Sánchez, Víctor Manuel Soto-Hermoso, Bernardo Requena

**Affiliations:** 1 HUMAN Lab, Sport and Health University Research Institute (iMUDS), University of Granada, 18016 Granada, Spain; vsoto@ugr.es (V.M.S.-H.); bernardorequena@icloud.com (B.R.); 2 FSI Sport Research Lab, 18016 Granada, Spain; albertofr_91@hotmail.com (A.F.-R.); t.dossantos@mmu.ac.uk (T.D.); jaimemorente@ugr.es (J.M.-S.); 3 Department of Sport and Exercise Sciences, Musculoskeletal Science and Sports Medicine Research Centre, All Saints Building, Manchester Campus John Dalton Building, Manchester Campus, Manchester Metropolitan University, Manchester M15 6BH, UK; 4 Manchester Institute of Sport 2.01, Manchester Metropolitan University, Manchester M1 7EL, UK; 5 Centre for Research, Education, Innovation and Intervention in Sport, Faculty of Sports of the University of Porto, Rua Dr. Plácido Costa, 91, 4200-450 Porto, Portugal; jneves@fade.up.pt; 6 Education and Research Department, Isokinetic Medical Group, FIFA Medical Centre of Excellence, 40132 Bologna, Italy; f.dellavilla@isokinetic.com

**Keywords:** knee injuries, injury prevention, movement quality, feasible interventions

## Abstract

Anterior cruciate ligament (ACL) is one of the most concerning injuries for football players. The aim of this review is to investigate the effects of exercise-based interventions targeting at reducing ACL injury rate or mitigating risk factors of ACL injury in adult football players. Following PRISMA guidelines, a systematic search was conducted in CINAHL, Cochrane Library, PubMed, Scopus, SPORTDiscus and Web of Science. Studies assessing the effect of exercise-based interventions in ACL injury incidence or modifiable risk factors in adult football players were included. 29 studies evaluating 4502 male and 1589 female players were included (15 RCT, 8 NRCT, 6 single-arm): 14 included warm-up, 7 resistance training, 4 mixed training, 3 balance, 1 core stability and 1 technique modification interventions. 6 out of 29 studies investigated the effect of interventions on ACL injury incidence, while the remaining 23 investigated their effect on risk factors. Only 21% and 13% studies evaluating risk of injury variables reported reliability measures and/or smallest worthwhile change data. Warm-up, core stability, balance and technique modification appear effective and feasible interventions to be included in football teams. However, the use of more ecologically valid tests and individually tailored interventions targeting specific ACL injury mechanisms are required.

## 1. Introduction

Football (soccer) is one of the most popular sports, with more than 260 million players around the world [1]. Football is also a sport exposed to a high risk of injury, considering that the overall injury incidence is 6.6 injures per 1000 players hours [2]. Given that being exposed to a high number of injuries reduce the chances to sporting success [3], injury management (i.e., mitigation and maximising player availability) is one of the most concerning issues in football clubs. Specifically, lower injury incidence rates has been correlated to superior performance (i.e., higher league position, more games won, more goals scored, greater goal difference and total points) in professional football [4], while injuries that cause a high injury burden (i.e., those requiring a high number of days lost, such us ligament sprains and joint injuries to the knee and the ankle) are more likely to impact negatively on team performance [5]. At the team level, the performance decrement associated to a high injury incidence can lead to losses of ~£45 million per season, on average, in English Premier League teams [6].

For the football player, one of the most concerning injuries is the anterior cruciate ligament (ACL) injury given its devastating consequences, such us the increased risk of developing early posttraumatic knee osteoarthritis [7,8], or the high rate of reinjuries to the graft or the opposite knee [9,10,11,12,13]. Furthermore, in professional football, only 60% of players who ruptured their ACL competed at the highest level 5 years later [14]. Additionally, ACL injuries in football causes, on average, an injury burden of 29.8 days per 1000 h of exposure [15]. Even though the ligament injury rate in European male professional football have decreased during the 2000s [16], the ACL injury rate showed no declining trend [17]. Therefore, efforts aimed at reducing the rate of ACL injury in football appear to require further development, being non-contact or indirect contact injuries the specific focus, as they correspond to the 88% of all ACL injuries [18] and could be targeted by injury risk mitigation programs [19]. Consequently, these non-contact or indirect contact ACL injuries will be the focus of this systematic review.

ACL injury is not an injury with a high incidence, with an average of 0.43–0.60 in-juries per team per season in football professional teams [17,20]. As a result of the low number of ACL injuries suffered per season, some potentially effective interventions could not be able to reach significance when rate is used to evaluate their efficacy [21,22]. As an alternative, in the purpose of reducing ACL injuries, intervention programs could be also developed with the aim of modifying risk factors of ACL injury [23], and efficacy could be assessed by measuring changes in proxy factors (i.e., surrogates of injury) [24,25]. Specifically, the identification of modifiable risk factors would increase the potential for screening athletes at higher risk and targeting interventions to address the specific mechanisms that increase ACL injury risk [26,27]. In football, ACL injuries mostly occur during cutting actions [18,28,29] with visual observational analyses confirming a mechanism of knee valgus, abducted hip, flat and externally rotated foot, and ipsilateral trunk tilt and contralateral rotation [18]. These aberrant movements have been shown to increase the multiplanar knee joint loads and, hence, increase the ACL load [30,31]. Additionally, neuromuscular factors have been shown to have implications for ACL injury [23,27]. In this sense, different metrics of hamstrings-to-quadriceps (H/Q) ratio could potentially provide useful information regarding the load that the ACL is assuming, as coactivation of hamstrings and quadriceps could protect the knee against anterior shear forces at the tibia [32]. Furthermore, balance ability could have an influence in ACL injuries, given the positive contribution of a higher hamstrings, hip and trunk muscle activation in supporting and reducing knee joint loadings [33]. Although findings are still inconclusive, movement quality and competency deficits may be further linked to greater joint loads [26]. Other risk factors such as restricted ankle or hip mobility could predispose the knee higher loads and increase the risk of ACL injury [34,35]. Therefore, some of these biomechanical, neuromuscular and physical capabilities could be potentially targeted with the purpose of modifying the risk factors of ACL injury [36].

To date, several systematic reviews have been published in the field of ACL injury prevention in athletes [21,23,37,38,39,40,41]. The last research in football players is the work published by Grimm and collaborators [21], where only randomized-controlled trials (RCT) in which ACL injury incidence was reported were included. Even though RCTs are considered level 1 of evidence, they are usually difficult to implement in the “real-world” environment, and other study designs (i.e., non-randomized, NRCT) could be additional sources of evidence used by practitioners to guide their practice and provide recommendations to their athletes [42]. On the other hand, at the best of our knowledge, the most recent systematic review that took into consideration the effect of preventive programs in both ACL injury incidence and risk factors of ACL injury in football players was conducted 12 years ago, by Alentorn-Geli et al. [23]. However, given that football has evolved [43] and new strategies to screen injuries have been developed in recent years [44], further research examining these new practices are required. Therefore, the aim of the present study is to systematically review the effects of exercise-based interventions on ACL injury rates and incidence or mitigating risk factors of ACL injury in adult football players. Thus, the review questions are summarised as follows: which exercise strategies are proposed to effectively reduce the incidence of ACL injuries and/or mitigate modifiable risk factors of ACL injury in football players? Are exercise-based training interventions feasible to implement in the context of the football player?

## 2. Materials and Methods

The systematic review was conducted following the guidelines of the Preferred Reporting Items and Meta-analysis [45]. The protocol for this review was registered at PROSPERO (ID = CRD42020205669). 

### 2.1. Search Strategy

The same systematic search was performed in CINAHL, Cochrane Library, Pub-med, Scopus, SPORTDiscus (EBSCO) and Web of Science. The following search strategy and key words were used, adapted for each database to limit the number of entries which could potentially be of interest (i.e., title, abstract and key words in Scopus, SportDiscus and CINAHL and all fields Cochrane, Pubmed and Web of Science): (ACL OR “anterior cruciate ligament”) AND (soccer OR football*) and (intervention OR pro-gram OR programme OR training OR modif* OR prevent* OR reduc* OR exercise OR correct*). The search was performed at January 2021, there were no restrictions for year of publication and only English language published studies were reviewed. Reference lists of the included studies were also searched to identify potentially missed, relevant studies.

#### 2.1.1. Eligibility Criteria

All types of exercise-based interventional studies (i.e., RCT and NRCT) aiming at both reducing ACL injury incidence and mitigating modifiable risk factors of ACL injury were included, regardless of its study design. Given the current limitations of study designs and the inappropriate methods used when aiming at establishing risk factors of injuries [22], any outcome variable that has previously been associated to ACL injury in the literature was considered and discussed according to the strength of its association (Table 1). The PICOS methods was used to set the criteria for study inclusion (Table 1).

#### 2.1.2. Study Selection

**Figure 1:**
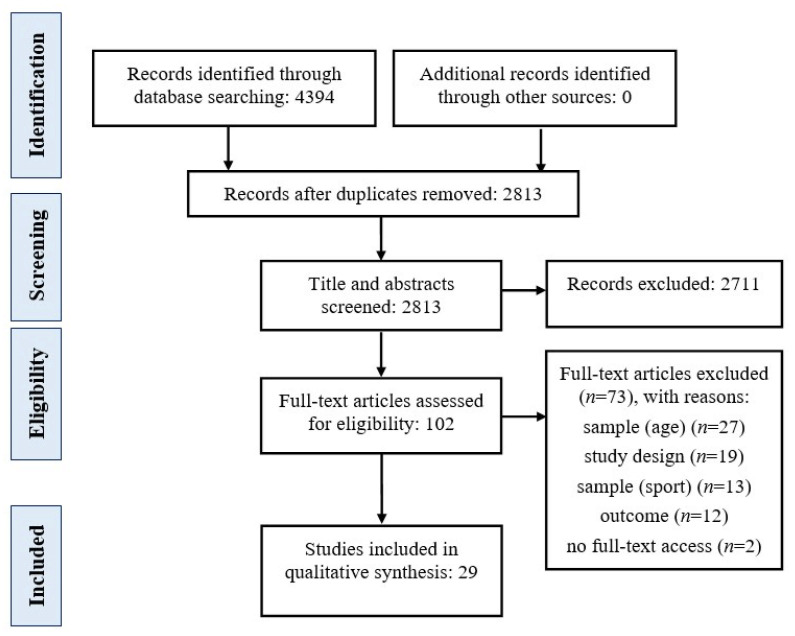
PRISMA flow diagram for the depiction of the overall process.

Two authors (JOJ and AFR) independently performed the study selection based on title and abstract screening. Full-text of those studies in which there were not absolute evidence for their exclusion, were retrieved and further analysed. Those cases where a discrepancy existed were resolved by an in-depth discussion between the authors (JOJ and AFR). If disagreements persisted, they were solved by a third author (TDS). PRISMA flow diagram for the description of the overall process is depicted in Figure 1. 

#### 2.1.3. Data Extraction 

Data of the included articles were subsequently extracted in an Excel spreadsheet in the same way that literature search was performed. The following data was extracted from each study: title, author(s), publication year, participant sex, participant age, number of participants in experimental and control groups, participant level, compliance rate, number of dropouts, supervisor of the program, type, contents and characteristics of the intervention, comparison group, outcome of interest, reliability data, smallest worthwhile change and changes in the outcome measures of interest from baseline to following the intervention. If the case of data elements of interest were missing or unclear, the study authors were contacted for clarification. In those studies in which no effect sizes (ES) were reported, the ES were manually calculated (ESc) from the extracted data using the corrected Hedges’g proposed by Turner et al. [47]. The scale proposed by Hopkins et al. [48] was used for interpretations of the magnitude of results, whereby the magnitude of ES was considered as trivial (≤0.20), small (0.20–0.59), moderate (0.60–1.19), large (1.20–1.99), or very large (≥2.00).

### 2.2. Risk of Bias Assessment

Risk of bias for RCTs was assessed using the Version 2 of the Cochrane Tool for assessing risk of bias in randomised trials (RoB 2) [49]. In the case of NRCTs, the tool used was the Risk of Bias in Non-Randomized Studies of Interventions tool (ROBINS-I) [50].

The above tools for assessing risk of bias are domain-based evaluation tools that are currently the frequently and preferred method to assess the credibility of study findings over quality checklists and quality scales [51]. The RoB 2 is a domain-based risk of bias assessment tool rigorously structured into five bias domains: (1) bias arising from the randomisation process, (2) bias due to deviations from intended interventions, (3) bias due to missing outcome data, (4) bias in measurement of the outcome, and (5) bias in selection of the reported result [49]. Since the aim of the review was to investigate the effect of assignment to the intervention at baseline instead of the effect of adhering to it (i.e., effectiveness vs efficacy), an intention-to-treat analysis was considered. For risk of bias assessment of cluster-RCT, a supplement of the RoB 2 proposed by the same authors was used. Depending on the judgments in each of them, an overall risk of bias for the result is established. The ROBINS-I has been designed in order that confounding factors and co-interventions that may potentially lead to bias in NRCT could be identified [50]. Previous injury, level, age, sex and exposure to training and competitions were considered confounders as they can influence the risk factors of ACL injury [23,27]. 

Two authors independently performed the RoB 2 and ROBINS-I (JAN and JMS) tools. After agreements and disagreements were discussed, in cases in which there persisted any disagreement, a third author (JOJ) was consulted for the final decision.

## 3. Results

### 3.1. Study Selection

Details of the search results are represented in Figure 1. The systematic search performed through the six previously mentioned databases provided 4394 records which were gathered in Endnote X9 software. After duplicates were identified and removed, 2813 records remained, reducing to 102 following screening by title and abstract. After full-texts were retrieved and further analysed, 29 studies met the eligibility criteria and were included in the systematic review. One study was excluded because, although the proposed programme consisted of 3 weeks of preventative training plus 5 months of maintenance, no information was reported regarding the maintenance phase [52]. Therefore, it is not possible to determine if this part targets the same components than the previous phase, and then, the programme did not reach the minimum 4-week duration established as reference criteria. Reasons for exclusion are detailed in Figure 1. No study of the references lists met the eligibility criteria.

### 3.2. Characteristics of Included Articles

The 29 studies included in the systematic review investigated the effects of exercise-based interventions on either reducing the incidence of ACL injury (6 studies) [53,54,55,56,57,58] or mitigating risk factors of ACL injury (23 studies) [59,60,61,62,63,64,65,66,67,68,69,70,71,72,73,74,75,76,77,78,79,80,81], where a sum of 6091 adult (male: 4502, female: 1589) football players were evaluated. 

The study designs used were 11 parallel RCT [60,62,63,64,65,68,69,73,78,80,81], 4 cluster RCT [53,56,57,58], 8 NRCT [54,55,59,66,67,72,75,79] and 6 single-arm studies [61,70,71,74,76,77]. 10 out of 15 RCT studies implemented at least one of the following four different preventative warm-up (WU) programmes (Table 2): (i) FIFA 11 [80], (ii) FIFA 11+ [56,57,58,60,63,73,78], (iii) Prevent injury and Enhance Performance (PEP) Program [53], and (iv) core stability training [65]. Additionally, the other 5 studies implemented resistance training (RT)- [62,64], balance- [68,69] or mixed training (MT)-based interventions [81]. Four out of these 15 RCTs were evaluated through its ability to reduce contact, non-contact or overall ACL injury incidence [53,56,57,58], while the other 9 were evaluated through its effect on different ACL injury risk factors, such as different measures of static and dynamic balance ability [60,63,68,69,73], H/Q ratios [62,64,65,80], biomechanics of dynamic tasks [65,73,80], Functional Movement Score (FMS) [78,81], and ankle ROM and symmetry in hop tests [60]. Only three studies reported reliability measures of their outcome data [65,68,78]; one study reported the smallest worthwhile change (SWC) [73]; nine studies adequately described who supervised the programme [53,56,57,60,64,65,73,78,81]; and five studies provided the compliance rate [53,56,58,62,80].

Regarding the 8 NRCT (Table 3), two studies implemented a WU-based intervention [54,59], 3 a RT-based intervention [67,72,79], one a balance-based intervention [75], 1 a technique modification (TM)-based intervention [66] and one a MT-based intervention [55]. The outcome of interest was ACL injury incidence in 2 studies [54,55], while ACL risk factors investigated were total, dynamic balance [75], H/Q ratio and bilateral strength differences [67,72,79], and biomechanics of dynamic tasks [59,66]. Only two studies reported reliability measures of their outcome data [59,66]; two the SWC [59,66]; five stated who was the supervisor of the programme [54,55,59,66,79]; and one the compliance rate [66].

**Figure 2:**
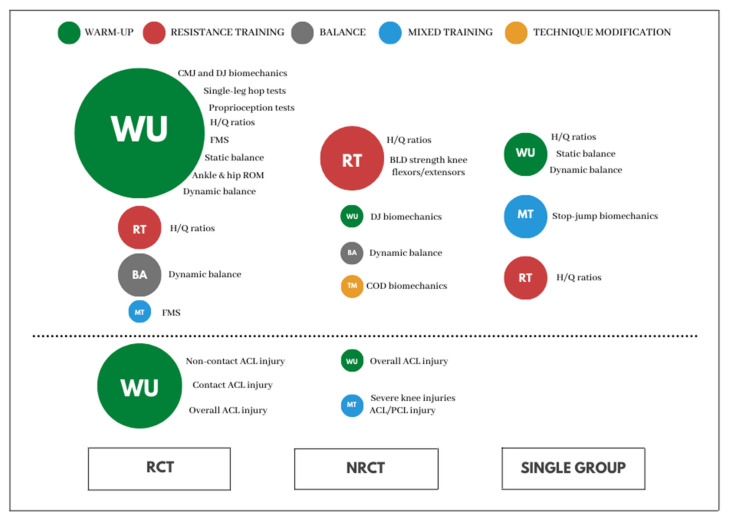
Representation of the different exercise-based interventions used to target both ACL injury risk mitigation (upper panel, over the dotted line) and ACL injury reduction (lower panel, under the dotted line), for each study design. The size of the circumferences represents the number of studies including the designated intervention (1 is the smallest, 6 is the biggest). To the right is the outcome measures used to evaluate their effectiveness. ACL—anterior cruciate ligament, BA—balance-based interventions, BLD—bilateral strength differences, CMJ—countermovement jump, COD—change of direction, DJ—drop jump, FMS—functional movement score, MT—mixed-training-based interventions, NRCT—non-randomised studies, PCL—posterior cruciate ligament, RCT—randomised-controlled trials, RT—resistance training-based interventions, TM—technique-modification-based interventions, WU—warm-up-based interventions.

Finally, the 6 single-arm studies with no CG investigated the effect of RT-based [71,74], MT-based [70,76] and WU-based interventions [61,77] on H/Q ratio [61,71,74], different measures of balance ability [77], or biomechanics of dynamic tasks [70,76] of ACL injury, with no study examining its effect on ACL injury incidence (Table 4). None of the single-arm studies reported directly measured reliability data or the SWC. Two studies reported compliance rate [61,70], and two reported the supervisor of the programme [70,76]. In Figure 2, a depiction of the different exercise-based interventions used to target both ACL injury risk mitigation and ACL injury reduction, for each study design, is presented.

### 3.3. Risk of Bias in Individual Studies

A summary of the risk of bias in both parallel and cluster RCT at the different domains level of the RoB 2 tool are displayed in Supplementary Table S1 and Figure S1. Only one study was reported to be at low overall risk of bias [73], while 4 present some concerns [62,65,78,80], and 10 are at high risk of bias [53,56,57,58,60,63,64,68,69,81]. Regarding the 8 NRCT, 6 of them were classified as critical risk of bias [54,55,59,67,72,79], while two were classified at moderate risk of bias [66,75] (Supplementary Table S2 and Figure S2). Single-arm studies will be only discussed as potentially feasible implementations whose effectiveness should be further investigated. Therefore, no risk of bias analysis was conducted. A detailed explanation of this section is included in the Electronic Supplementary Material.

### 3.4. Results of Individual Studies

Among the 29 included studies in the review, 6 studies evaluated the effect of exercise-based programs on ACL injury incidence. Four studies were RCT, in which three warm-up-based interventions (i.e., PEP Program [53], and FIFA 11+ [56,57]) found a protective effect on ACL injury incidence, while one in which part 2 of FIFA 11+ was performed at the end of the training showed no differences against the traditional FIFA 11+ [58]. Two studies were NRCT, one including FIFA 11+ [54] and the other a MT-based interventions [55], both of which demonstrated no protective effects on ACL injury incidence. 

23 studies evaluated the effectiveness of exercise-based programs on different risk factors of ACL injury. 13 studies were RCT. Six studies used warm-up-based interventions (i.e., FIFA 11+), four of them showing positive results in improving different metrics of static and dynamic balance [60,63,73], GRF asymmetries in countermovement jumps and conventional H/Q ratios at 1.05 and 3.14 rad/s [65], and five showing no effect on balance [60,73], ankle and hip ROM, asymmetries in hop tests [60], frontal plane angles during a DVJ [80], conventional H/Q ratios at 60 and 240 deg/s [80] and Functional Movement Score (FMS) [78]. Two balance-based interventions showed positive results on different metrics of balance [68,69]. Two RT-based interventions found positive [64], and no [62] improvements in different metrics of H/Q ratios. One MT-based intervention [81] was effective at improving some metrics of the FMS. 6 additional studies were NRCT. One warm-up intervention did not improve drop-jump biomechanics [59]. Three RT-based interventions were effective in improving H/Q ratio [67,72,79], while one was effective at reducing bilateral between-limb differences in isokinetic knee extensors and flexors strength [67], and other was not [79]. One balance-based intervention was effective at improving balance metrics [75]. One TM-based intervention was effective in improving COD movement quality and performance [66]. 

6 studies were single-arm studies. Two used warm-up-based interventions that were effective at improving H/Q ratios [61] and balance [77]. Two used RT-based interventions, effective at improving functional, but not conventional H/Q ratio [71,74]. Two used MB-based interventions, effective at improving stop-jump task biomechanics [70,76].

When analyzing the findings of individual studies according to their risk of bias (Supplementary Tables S1 and S2), the only study at low risk of bias found that FIFA 11+ was effective at improving time-to-stabilization during a single-leg landing task (−1.8%), but not at improving core stability or SEBT [73]. Two studies at moderate risk of bias also found no effect of FIFA [80] and FIFA 11+ [78] on H/Q ratio and knee valgus angle during a countermovement jump, and in FMS, respectively. Contrasting to these findings, other high-risk studies investigating the FIFA 11+ found positive effects of the programme on both ACL injury incidence [54,57] and several risk factors of injury [59,60,63] (Table 3 and Table 4). Other studies at moderate risk of bias found positive effects of RT-based interventions on functional H/Q ratio during fatigued conditions [64] but not in conventional, unfatigued H/Q ratio [62]. Dello Iacono et al. [65] found a positive influence of a core stability training on conventional H/Q ratio and in GRF asymmetries during one-leg countermovement jumps (CMJ). Finally, the study carried out by Dos’Santos et al. [66] showed promising results, as their TM-based programme was effective at improving performance and movement quality during COD. The rest of studies were considered to be at high or critical risk of bias. Therefore, their findings must be considered with caution.

## 4. Discussion

To our best knowledge, this is the first systematic review which investigates the effects of exercise-based interventions on ACL injury incidence and risk factors in football players from that of Alentorn-Geli et al. [23] published 12 years ago. The summary of findings and their implications will be summarised in the following sections: (Section 4.1) Effects of exercise-based interventions on ACL injury incidence; (Section 4.2) Effects of exercise-based interventions on risk factors of ACL injury; and (Section 4.3) Potentially feasible interventions from single-arm studies. Following this, a discussion of the importance of different risk factors and practical applications will be provided.

### 4.1. Effects of Exercise-Based Interventions in ACL Injury Incidence

#### 4.1.1. Evidence from Randomised-Controlled Trials

Four cluster-RCTs investigated the effects of warm-up-based interventions on ACL injury incidence showing varied results (Table 1), all of which were classified as high risk of bias (Supplementary Table S1 and Figure S1). Gilchrist et al. [53] and Silvers-Granelli et al. [56,57] found that PEP Program and FIFA 11+, respectively, could effectively reduce the ACL injury incidence. In the case of FIFA 11+ there was a 46.1% reduction of injuries in the IG, with 70 number-needed-to-treat (i.e., 70 athletes needed to be treated to prevent 1 ACL injury). Even though a similar reduction (i.e., 43.8%) in injury rates was found in female athletes who participated in neuromuscular training (FIFA 11+ could be considered neuromuscular training) [82], number-needed-to-treat data should not be compared since it is time-dependent [83] and different designs have been used for its calculation (i.e., RCT of one competitive season vs. systematic review with different time windows studies) [56,84]. These findings are in line with a systematic review concluding that FIFA 11+ is effective at reducing injuries in football players [84]. In spite of this, surprisingly only 10% of FIFA’s member governing bodies associations have implemented the programme with researchers highlighting a low compliance with the 11+ [85,86]. This low adoption and compliance could be explained, among others, by the time required to complete (~20–25 min), boredom associated with the programme [85], and the soreness and fatigue caused by exercises (i.e., Nordic curls) contained in Part 2 [85,86]. To prevent these issues and improve compliance, Whalan et al. [58] proposed a rescheduled FIFA 11+, in which parts 1 and 3 were performed during the warm-up, while part 2 was performed at the end of the training. However, no differences against traditional FIFA 11+ were observed. Given that effectiveness partly relies on the compliance and implementation of any prevention programme [87], it remains unknown if FIFA 11+ could effectively reduce ACL injury rate in the “real world”, particularly at the sub-elite and community level.

#### 4.1.2. Evidence from Non-Randomised Trials

The two NRCT investigating the effect of exercise-based intervention on ACL injury rate [54,55], both classified as critical risk of bias, did not demonstrate any reduction in ACL injury rate (Table 2), even though one MT-based programme was effective at reducing severe knee injuries [55]. The fact that this reduction was achieved within a short duration MT-based protocol per session (i.e., 12 min approx.) warrants further investigation through higher quality NRCT or RCT. In Table 5, an overview of the findings is provided for the effects of the exercise-based interventions on ACL injury incidence (Section 4.1).

### 4.2. Effects of Exercise-Based Intervention in Risk Factors of ACL Injury 

#### 4.2.1. Evidence from Randomized-Controlled Trials

6 out of 11 studies investigating the effects of interventions on risk factors of ACL injury were warm-up-based interventions (Table 1). The most frequently warm-up administered was the FIFA 11+. Some studies reported that the FIFA 11+ was effective at improving different surrogates of ACL injury risk [60,63], while others found no differences compared to a CG [63,73,78,80]. Notably, the only RCT in the present systematic review assessed at low risk of bias found no meaningful differences between the FIFA 11+ and CG in SEBT following a 9-week intervention, although an improvement, albeit small, in time-to-stabilisation in IG (−1.8%) was observed [73]. However, the practical implication of such small change into overall ACL risk of injury remains unknown. Although the FIFA 11+ has been shown to decrease ACL injury rate, the controversy of the findings (some studies reporting positive while others no effects for a given component) makes difficult to elucidate the mechanisms by which the programme may be effective at decreasing the incidence [87]. The effectiveness of a warm-up-based stability training in both H/Q ratio and asymmetry in GRF during CMJ [65] requires further research to corroborate these findings. Additionally, of note, changes in H/Q ratio should be considered with caution, as they can be obtained through different changes in the hamstrings and quadriceps musculature (i.e., numerator and dominatory; for example, a reduction in quadriceps strength could improve the ratio, but probably not the risk of injury), thus the constituent components of any ratio should always be examined.

Two studies included a balance-based intervention in order to improve balance and stability [68,69], both showing a positive effect. Balance training has been proposed to be an effective component in both reduction of ACL injury rate and mitigating the predisposing factors [27,88]. Recently, balance training has been considered an effective strategy to mitigate risk factors of ACL injury during COD [89], potentially attributable to positive changes in hamstring, hip and trunk muscle activation, which supports and reduces knee joint loads [33]. However, the high frequency of training needed (i.e., 6 times a week) [68] and the small magnitudes of the effects [69] question the effectiveness and feasibility of these interventions. Additionally, the absence of clear differences between unstable and stable surfaces [69] in balance are in line with previous works [90], although the potential reductions in strength and power application as a consequence of training on unstable surfaces should be considered when they are implemented [91].

A 7-week RT-based intervention showed promising results improving functional H/Q ratio in both fatigued and unfatigued states [64]. RT has been commonly proposed to be a critical component of successful injury prevention programmes in football and is central for facilitating positive tissue adaptations and robustness [92]. Among its potentially beneficial effects include improved coordination, enhanced technique in different activities, reduction of potentially hazardous joint loads, and improvement of the psychological perception of high-risk situations seem to be possible mechanism for reducing the risk of suffering acute injuries, such us ACL tear [93]. However, the requirements of gym-based equipment and the high weekly volume (i.e., >20 min performed 3/week) [64] makes difficult its inclusion into most football training contexts.

Of note, it must be highlighted that only three out of nine studies directly measured the reliability of their data [65,68,78], while one acknowledges other reliability data [60,64] and four studies did not report any reliability data at all [63,69,73,80]. Furthermore, only one study [73] established the SWC. Taken collectively, the findings of the studies must be interpreted with caution, because it is uncertain whether the training-induced changes exceeded the measurement error, and therefore casts doubt whether they were “true” or “real” findings.

#### 4.2.2. Evidence from Non-Randomized Trials

The most common intervention investigated in a NRCT design were RT-based interventions, showing effectiveness at improving H/Q ratio and bilateral differences in peak torque flexor/extensor ratios [67,72,79], in line with the previously mentioned advantages that RT possesses in relation to decreasing the risk of injury [92,93]. However, the serious methodological issues present in their designs resulted in critical risk of bias classification.

A balance-based intervention, similarly to which was found in RCT, found positive effects of a football-specific balance programme on static balance assessments [75], which aligns with the literature evidence suggesting that balance training as a component [87], and specificity as a feature of prevention programmes [94] may positively influence in the risk of injury. 

Two studies evaluated the effectiveness of their interventions on lower-body biomechanics of dynamic tasks that try simulate the mechanisms of ACL injury (i.e., landing/COD) [59,66], showing no meaningful effect of FIFA 11+ on peak knee flexion angle during a drop vertical jump [59] and an improvement of a TM-based intervention on COD performance and quality of movement [66]. The findings of the latter intervention are appealing because of several factors: (i) it is one of the only two NRCT studies at moderate risk of bias; (ii) it only requires two 20-min sessions a week which can easily implemented on the field; (iii) both performance and injury risk are improved which is likely to improve athlete and coach adherence and compliance; and (iv) the results were practically meaningful (compared against SWC and SDD). In the absence of robust risk factors of ACL injury [22], it seems to be worthwhile developing training modalities that aim at reducing potentially high-risk postures associated with ACL injury mechanisms [95], where COD has been widely shown to be the main injury mechanism [18,20,29], and which are effective both from a performance and injury perspectives, as they will be more easily adopted by coaches and practitioners [31].

Similar to the aforementioned RCTs, it must be highlighted that only 2 out of 6 studies reported reliability and SWC data [59,66], while the other 4 failed to did not report any information related to reliability measures [67,72,75,79]. Therefore, again, it is difficult to determine the practical relevance of the results reported. In Table 5, an overview of the findings for the effects of the exercise-based interventions on risk factors of ACL injury is provided.

### 4.3. Potentially Feasible Interventions from Single-Arm Studies

Single-arm study characteristics are displayed in Table 3, although due to the previously mentioned limitations as a consequence of not containing a CG, interventions which could be easily implemented in any context (i.e., no sophisticated equipment and so much time required) will only be discussed. Some of the single-arm studies proposed interventions required machine and free-weights [70,71,76] which is equipment not easily available in any training facilities, or they correspond more to full training programmes (i.e., 60-min RT sessions twice a week, plus 60-min field conditioning session twice a week) than preventative interventions [70,76]. Regarding only those potentially feasible in the field, two preventative programs effective at improving muscle activity (i.e., part 2 of FIFA 11+ [77]) or H/Q ratio (i.e., RT-based [74]) may merit further research through multiple-arm, RCT. However, until then, their possible influence on ACL injury risk remains, at best, speculative.

### 4.4. Do All Risk Factors Established Elsewhere in The Literature Equally Contribute to ACL Injury?

Many risk factors have been proposed to be related to ACL injury throughout the years [27,30,96,97,98,99,100,101]. Among them, it is modifiable risk factors which have risen in interest given that they can be targeted by preventative programmes [102]. Neuromuscular (e.g., antagonist-agonist relationships, muscle activation, decreased co-contraction, decreased proprioception) and biomechanical (i.e., ankle, knee, hip and trunk movements in three planes of motion) deficits have been frequently proposed as main modifiable risk injuries of ACL injury [23,96,99], also specifically in football players [23]. Although some of these factors have been proposed to be related ACL injury [103,104], others have presented opposing results (e.g., no relationship between FMS and injury) [105].

With the final purpose of reducing the likelihood of suffering an ACL injury, screening tests should be validated, in a three-step process [22]: (i) a strong relationship between a marker from a screening test and injury risk must be found in prospective studies, (ii) the test properties of the marker must be validated in relevant populations, with appropriate statistic techniques, and (iii) an intervention programme targeted to athletes at high risk must be more beneficial that the same program targeted to all the athletes. Nevertheless, even these prospective studies also have limitations, such us the fact of relating the determined marker with an injury that occurs weeks or months later [106].

In football players, ACL injury mechanisms have been widely established [18,28,29,107]. In the two most recent visual observational analysis studies of ACL injury mechanisms in professional players (male and female) [18,28], it was concluded that performing a COD while pressing or tackling was the main common situation, where ipsilateral trunk tilt and contralateral rotation, abducted hip, dynamic knee valgus and flat and externally rotated foot were the most frequently observed mechanisms [18,28]. Other studies have also found COD and landings to be the main non-contact ACL injury mechanisms in football [29,96] and American football [107]. Therefore, in the absence of strong evidenced risk factors to be targeted, evaluating movement quality and identifying biomechanical and neuromuscular deficits during potentially high-risk maneuvers (i.e., landings and CODs) can provide important information regarding an athlete’s “injury risk profile” [108,109]. 

Three-dimensional motion analysis is the gold standard for evaluating biomechanical variables; however, they require high-cost equipment and are time-consuming to be applicable in football [44]. To overcome these limitations, several cost-effective qualitative field-based screening tools have been developed, such us the landing error scoring system (LESS) [110], tuck jump assessment (TJA) [111], qualitative analysis of single leg loading (QASLS) [112], CMAS [109] or the 2D video analysis scoring system of 90° CODs [113] to evaluate risk of ACL injury. Since these assessments have been developed to identify biomechanical and neuromuscular control deficits similar to the direct mechanisms of ACL injury (i.e., landings, cuttings), and they can be simply evaluated in the field context, future research is necessary that investigates the effect of exercise-based training interventions on biomechanical and neuromuscular control deficits (movement quality) during landing and cutting field-based screening tests in footballers [22]. Furthermore, even though a better movement quality in common mechanisms of ACL injury could mitigate the risk of injury, few published prevention programs appear to expose athletes to the task-specific elements of the injury mechanisms [95], a field that warrants further research.

### 4.5. Practical Applications

Based on the findings of the systematic review, warm-up-based interventions such us FIFA 11+ or PEP Program seem to be effective at reducing ACL injury rates [53,56,57]. These aforementioned field-based methods can be easily performed at the community to elite level in football because they do not require sophisticated equipment, and generally take only 20 min. However, the mechanisms by which these interventions are effective remains unanswered from the findings of the present work, although from studies with preadolescent female football players, it can be speculated that it is effective at reducing knee valgus angles and moments during landings, probably due to the large volume dedicated to jump-landing technique [114,115]. Nevertheless, it appears to be a poor modality at inducing favorable changes in cutting technique, most likely to the low volume of cutting drills in the programme [114,115]. In the case of FIFA 11+, though, it presents some pitfalls which potentially limits its implementation in football teams despite its effectiveness in reducing injury rates. Additionally, it could be more appropriate and specific in relatively inexperienced players, particular at the community level, as this could be a sufficient training stimulus to elicit improvements in balance, strength, or NM control. Some core stability [65], balance [68], and TM interventions [66] have shown promising results, since they can potentially provide protective benefits in a feasible way (i.e., basic equipment, and no more than 20-min sessions, 5/week interventions). Conversely, some of the included RT-based interventions have been shown to be effective at targeting neuromuscular factors, although the interventions used makes difficult their translation to the real context. 

In order to reduce ACL injury rates, it is necessary that the proposed interventions are both effective and feasible (i.e., providing protective effects and easily implementable in the real context), and not only show efficacy in ideal conditions [116]. This can be achieved by developing shorter and more flexible prevention programmess that facilitates its inclusion in football teams [89]. There is a scarcity of RCT and NRCT investigating the effect of exercise-based interventions on movement quality tasks simulating the main mechanisms of ACL injury (i.e., landing and COD), an area which warrants further research. Additionally, none of the interventions have been individually designed to target the identified specific high-risk factors of the football player (e.g., knee valgus, poor trunk control). Although general interventions would be more easily implementable, the more feasible individualization in elite teams also warrants further research. Finally, despite the effectiveness of mixed multicomponent program as a way to reduce ACL injuries [117], none of the included interventions included the main components in the way in which have been previously recommended [27,118]. The use of these multicomponent programs, which could potentially improve also other physical qualities, would improve the external validity of these interventions. 

### 4.6. Limitations

Our systematic review is not free of limitations. Firstly, studies including team sports athletes other than football players were excluded from the analysis. However, it could be argued that some interventions could be useful for different team sports. Given the uniquely characteristics of the different sports, including football, the aim of the present systematic review was to gather only those specifically carried out in football players. Secondly, a meta-analysis was not conducted, although a high number of studies were included. The two main reasons were: (i) there were three types of different study designs which should not be mixed; and (ii) within each type of study designs, interventions, comparators and outcomes of interests were too heterogeneous to conduct a proper meta-analysis. Additionally, given the low number of ACL injuries per team per season, most of the study interventions aiming at reducing ACL injury incidence found difficulties to reach significance, hence masking their potentially protective effects. Although expanding the literature search to overall knee injuries would solve this problem, the variability of injuries, with their associated specific risk factors, would make it hard to provide specific recommendations. Finally, when analysing ratios as potential outcomes for injury risk mitigation (i.e., H/Q ratio), it should be considered that they may not change because of both components increasing (i.e., strengthening of knee flexors/extensors) or decreasing (i.e., weakening), which would drastically determine the interpretation of the change. However, these sub-components analyses are not always performed in research investigations.

## 5. Conclusions

Our findings revealed that some exercise-based strategies could be potentially effective at both reducing ACL injury rate and mitigating risk factors of ACL injury in adult footballers. Warm-up-based interventions such us FIFA 11+ or PEP Program appear effective and feasible in the context of football teams across community to elite levels, although the mechanisms by which they are effective are not well understood. Core stability, balance and TM-based interventions appear to be better options to include as preventative programmes, as they are feasible and meaningfully decreased risk factors of ACL injury. Other included RT-based interventions have shown protective effects in neuromuscular deficits related to ACL injury (i.e., H/Q ratio), although their time and equipment required makes difficult to potentially be implemented in football teams. Consequently, for future work, improvements in risk of bias frequently observed in RCT and NRCT is required, as well as further research into the effect of potentially effective and feasible interventions in movement quality in relation to sport-specific task similar to the main mechanisms of ACL injury, such as landing and cutting tasks. More research analyzing the effect of interventions targeting individual risk factors of ACL injury are also needed. Finally, considerations for future research are discussed in Table 6.

## Figures and Tables

**Table 1:** Criteria for inclusion according to the PICOS method.

Definition	Inclusion Criteria	Exclusion Criteria
Population	Adult (≥16 and ≤40 years old) football players (i.e., Association football) of any level Both male and female Not having suffered a severe injury the previous 2 years	Studies including different cohorts of athletes apart from football players (e.g., basketball, volleyball, handball) in which no sub-analysis by sport was performed
Intervention	Exercise or training-based interventions lasted at least 4 weeks, performed twice a week	Interventions performed with exogenous modalities (i.e., bracing, taping, etc.) or those exercise-based interventions lasting less than 4 weeks.
Comparator	Control group data if available (although not necessarily)	No exclusion criteria by comparator
Outcome	Either contact or non-contact ACL injury incidence or rate of injury Test measurements evaluating any modifiable risk factor (i.e., potentially targeted by exercise-based interventions) previously reported to have an influence in ACL injury [23,26,27,46], such as biomechanical, neuromuscular and/or physical tests (e.g., biomechanics of landing or cutting actions, H/Q ratio, balance measures, ankle or hip range of motion, etc.)	Overall injury incidence not explicitly reporting ACL type injuries Test measurements evaluating non-modifiable risk factors (e.g., anatomical)
Study design	Randomised-controlled trials Non-randomised studies Single-arm studies	Systematic reviews, meta-analysis, conference papers, book chapters or studies published in languages other than English.

ACL anterior cruciate ligament, H/Q hamstrings to quadriceps.

**Table 2:** Characteristics of the randomised-controlled trials included in the systematic review.

Reference	Participants Level	Intervention	Comparator	Outcomes	Compliance Rate	Reliability/SWC	Results	Comments
Gilchrist et al. 2008 [53]	1435 female soccer player (IG = 583, age: 19.88 years; CG = 852, age: 19.88 years) NCAA Division I	**IG:** PEP Program **Duration:** 12 weeks **Frequency:** 3/week **Session duration:** <30 min **Training components:** Stretching, strengthening, plyometrics, agilities, and avoidance of high-risk positions depicted on a video. Replacement exercises to alleviate boredom.	CG: Their customary warm-up.	Contact ACL injury, and non-contact ACL injury rate per 1000 AE.	72%	NA	↓ ACL injury rate in practice, and non-contact ACL injury rate in those with history of past ACL injury and late in season in IG. ↔ ACL injury rate and non-contact ACL injury rate in IG and CG.	Supervision: Certified athletic trainer. 8 dropouts in IG. Low compliance rate in IG. Lack of control the drills executed and of the uses of the program.
Steffen et al. 2008 [80]	31 adolescent female football players (IG = 17, CG = 14; age: 17.1 ± 0.8 years) Elite sport high school	**IG:** FIFA 11 **Duration:** 10 weeks **Frequency:** 3/week **Session duration:** ≈15 min **Training components:** 10 exercises focusing on core stability, neuromuscular control, eccentric hamstrings strength and agility.	CG: Regular warm-up (running and ball exercises)	Conventional H/Q ratio at 60 and 240 °/s and functional H/Q at 60 °/s. Frontal plane knee angles during CMJ and DVJ.	73%	NR/ NR	↔ No differences between groups in either H/Q ratios or valgus angle during CMJ and DVJ (*p* > 0.05)	Supervision: Project coordinator. Unbalanced groups. 2 dropouts in a small sample. Low power to detect differences.
Brughelli et al. 2010 [62]	28 soccer players (IG = 13, age: 20.7 ± 1.6 years; CG = 11, age: 21.5 ± 1.3 years). Professional	**IG:** Additional eccentric training **Duration:** 4 weeks **Frequency:** 3/week **Session duration:** 10–15 min **Training components:** 4–5 sets from 1–2 exercises, of 4 different eccentric exercises: Eccentric box drops, lunge pushes, forward deceleration steps, and reverse Nordic hamstrings.	CG: Regular field-based warm-up, but also training the Nordic hamstrings exercise (once a week, total of 2 sets of 6 reps)	H/Q ratio at 60 °/s	100%	Acknowledges another reliability data/ NR	↔ Q/H ratio at 60 °/s in both groups (*p* > 0.05)	Supervisor not specified. Drop of participants in CG (3). Low volume of additional training, and only 4 weeks.
Daneshjoo et al. 2010 [63]	36 male soccer players (IG1 = 12, age: 19.2 ± 0.9; IG2 = 12, age: 17.7 ± 0.4; CG = 12, age: 19.7 ± 1.6) Professional	**IG1:** FIFA 11+ **Duration:** 8 weeks **Frequency:** 3/week **Session duration:** ≈20–25 min **Training components:** (1) Running exercises; (2) strength, balance, muscle control and core stability; and (3) advanced running exercises. **IG2:** HarmoKnee **Duration:** 8 weeks **Frequency:** 3/week **Session duration:** ≈20–25 min **Training components:** (1) Warm up; (2) muscle activation; (3) balance; (4) strength; and (5) core stability	CG: Regular field-based warm-up	Error of proprioception test at 30, 45 and 60°. Distance excursion in SEBT. Time in the stork stand balance test, with open and close eyes.	NR	NR/ NR	↔ No significant difference between groups in either proprioception or static balance. ↑ Static balance with eyes opened in the 11+ (ES_c_ = 2.25, *p* = 0.043) and HarmoKnee (ES_c_ = 0.81, *p* = 0.011) and closed in the 11+ (ES_c_ = 2.78, *p* = 0.027; HarmoKnee (ES_c_ = 2.45, *p* = 0.022) in post- vs. pre-intervention. ↑ SEBT in the 11+ (ES_c_ = 0.82, *p* = 0.004) and HarmoKnee (ES_c_ = 0.91, *p* = 0.011) (time x group interaction: F = 3.767, *p* = 0.034).	Supervisor not specified.
Gioftsidou et al. 2012 [68]	38 male soccer players (age: 22.7 ± 3.5 years) 1st Greek division	**IG:** Balance training program **Duration:** 6 weeks **Frequency:** 3/week **Session duration:** ≈20 min **Training components:** Four different soccer-specific (controlling, passing, heading) balance exercises performed on a hemi-cylindrical board and on an hemi-spherical board.	CG: Standard soccer training	Total (SI), anterior-posterior (API) and medial-lateral (MLI) index. Maintenance time for the anterior-posterior (APM) and medial-lateral (MLM) movements.	NR	ICC = 0.67–0.80/ NR	↓ SI (ES_c_ = 0.67–0.70), API (ES_c_ = 0.65–0.74) and MLI (ES_c_ = 1.36–1.62) in both legs in IG. ↑ APM (ES_c_ = 1.71–3.02) and MLM (ES_c_ = 1.49–1.53) in both legs in IG	Supervisor not specified. No *p* values.
Impellizzeri et al. 2013 [73]	81 male soccer players (CG = 39, age: 23.3 ± 2.8; IG = 42, age: 23.7 ± 3.7 years) Amateur	**IG:** FIFA 11+ **Duration:** 9 weeks **Frequency:** 3/week **Session duration:** ≈20 min **Training components:** (1) Running exercises; (2) core and leg strength, balance and plyometric/agility; and (3) higher-speed running drills with cutting manoeuvres. The key element is promotion of proper technique.	CG: Traditional warm-up	SEBT. Time-to-stabilisation on a single leg during a jump-landing task. Unstable sitting posture test.	NR	SWD = 0,2*SD	↓ Core stability (8%) in CG. ↔ Core stability (1.5%) in IG. ↔ Time-to-stabilisation (1.5%) in CG. ↓ Time-to-stabilisation (−1.8%) in IG.	Supervisor: Fitness coach. Results rounded to decimals: Problematic to detect differences.
Silvers-Granelli et al. 2015 [56]	1525 male soccer players (IG = 675, age: 20.40 ± 1.66; CG = 850, age: 20.68 ± 1.46 years) NCAA Division I & II	**IG:** FIFA 11+ **Duration:** 1 competitive season **Frequency:** 3/week **Session duration:** ≈20 min **Training components:** (1) Running exercises that encompass cutting, COD, decelerating and proper landing techniques; (2) strength, plyometric and balance exercises that focus on core strength, eccentric control and proprioception; (3) running exercises.	CG: Typical soccer warm-up	ACL injury incidence per 1000 AE	Mean utilisation: 30.47 ± 12.16 sessions (considered moderate; total 18 games and 51/52 sessions)	NA	↓ ACL injury incidence rate (0.362/1000AEs vs. 0.085/1000AEs) in IG vs. CG. ↓ Likelihood of incurring an ACL injury (RR = 0.236 [0.193–0.93]; NNT = 70, *p* < 0.001)	Supervisor: Certified athletic trainer. Weeks of training not reported.
Dello Iacono et al. 2016 [65]	20 young male football players (IG = 10, age: 18.7 ± 0.67 years; CG = 10, age: 19 ± 0.063 years) Elite team national Israel league	**IG:** Core stability training **Duration:** 6 weeks **Frequency:** 5/week **Session duration:** ≈20 min **Training components:** The program consists of two parts: (1) improving balance and core stability, and (2) developing lower limb strength and neuromuscular control.	CG: Regular warm-up	H/Q conventional ratio at both 1.05 and 3.14 rad/s IA from GRF peak in a one-leg CMJ.	NR	95% limits of agreement: Isokinetic tests = −1.32–1.75 Jump tests = −2.12–1.96 ICC = 0.925–0.978/ NR	↑ H/Q ratio at 1.05 rad/s (ES = 0.61–0.75), and 3.14 rad/s (ES = 0.71–0.95) in both legs in IG. ↔ H/Q ratio at both velocities in CG. ↓ IA in IG (−71.4%, ES = 2.01). ↑ IA in CG (33.3%, ES = 1.28).	Supervisor: Researcher. Regular warm-up of the CG only consists of jogging, dynamic stretching and mobilisation.
Gonzalez-Jurado et al. 2016 [69]	18 male soccer players (IG = 9, age: 25,89 ± 3.85; IG2 = 10, age: 23.33 ± 3 years) 2nd Spanish division	**IG1:** Proprioceptive training on stable surface **Duration:** 5 weeks (5 phases of one week each) **Frequency:** 5/week (first 4 phases) and 3/week (last phase) **Session duration:** ≈5 min (estimated) **Training components:** Monopodal proprioceptive training exercises on a stable surface, adapted to football, and executed using a 4-station circuit.	IG2: Proprioceptive training on unstable surface Duration: 5 weeks (5 phases of one week each) Frequency: 5/week (first 4 phases) and 3/week (last phase) Session duration: ≈5 min (estimated) Training components: Monopodal proprioceptive training exercises on different unstable surfaces (soft mat, Freeman Dish, Fit-sit Platform and Dyn-air), adapted to football, and executed using a 4-station circuit.	Star Excursion Balance Test (maximum distance reached in 8 directions)	NR	NR/ NR	↑ Front left, Ant-Lat left, Lat right, Back right, and Ant-Med right (ES = 0.13–0.55) in IG1 and Front right and left, Ant-Lat left, Lat left, Post-Lat right, Post right and left, Post-Med right and left, Med right, and AntMed left (ES = 0.06–0.43) in IG2 ↑ AntMed right in IG vs. IG2 (intergroup analysis)	Supervisor not specified. Low sample size in each group. Daily training too short (5 min?).
Silvers-Granelli et al. 2017 [57]	1525 male soccer players (IG = 675, age: 20.40 ± 1.66; CG = 850, age: 20.68 ± 1.46 years) NCAA Division I & II	**IG:** FIFA 11+ **Duration:** 1 competitive season **Frequency:** 3/week **Session duration:** ≈20 min **Training components:** (1) Running exercises that encompass cutting, COD, decelerating and proper landing techniques; (2) strength, plyometric and balance exercises that focus on core strength, eccentric control and proprioception; (3) running exercises.	CG: Typical soccer warm-up	ACL injury incidence per 1000 AE	NR	NA	↓ ACL injury incidence rate (RR = 0.24 [0.07–0.81], *p* = 0.021) and non-contact ACL injury incidence rate (RR = 0.25 [0.06–1.15], *p* = 0.049) in IG vs. CG. ↔ Contact ACL injury incidence rate (RR = 0.21 [0.03–1.74], *p* = 0.148) in IG vs. CG.	Supervisor: Certified athletic trainer. Low number of ACL injuries. High amount of lost follow ups in the IG (100 players). Per protocol analysis.
Ayala et al. 2017 [60]	41 male youth football players (age: 16.8 ± 0.7 years) Amateur	**IG1:** FIFA 11+ **Duration:** 4 weeks **Frequency:** 3/week **Session duration:** ≈20–25 min **Training components:** (1) Running exercises; (2) strength, balance, muscle control and core stability; and (3) advanced running exercises. **IG2:** HarmoKnee **Duration:** 4 weeks **Frequency:** 3/week **Session duration:** ≈20–25 min **Training components:** (1) Warm up; (2) muscle activation; (3) balance; (4) strength; and (5) core stability	CG: Regular field-based warm-up	Y-Balance test. Ankle and hip ROM. Single hop for distance (asymmetry). Triple hop for distance (asymmetry).	NR	Acknowledges another reliability data/ NR	↑ Triple hop LSI score (very likely substantial difference [98%]), anterior distance (likely substantial difference [89%]), and posteriomedial distance (possibly substantial difference [60%]) in FIFA 11+ vs. CG ↔ No main effects in ankle ROM, LSI during single hop, posterolateral and composite score in FIFA 11 vs. CG and in all variables (possibly/likely trivial) in HarmoKnee vs. CG).	Supervisor: Trained rehabilitation specialist. Low sample of each group.
Delextrat et al. 2018 [64]	21 female soccer players (IG1 = 10, age: 21.8 ± 4.0; IG2 = 11, age: 23.7 ± 7.2) Amateur.	**IG1:** Strength endurance **Duration:** 7 weeks **Frequency:** 3/week **Session duration:** 10–15 min **Training components:** 6 sets of 12–20 rep progressing by decreasing the inter-set rest period (90 to 45s) of two hamstring strength exercises: (1) seated hamstrings curl, and (2) stiff-legged deadlifts.	IG2: Strength Duration: 7 weeks Frequency: 3/week Session duration: 20–25 min Training components: 6–10 sets of 6RM progressing by increasing load (80 to 100% of 6RM), with 3-min inter-set rest, of two hamstring strength exercises: (1) seated hamstrings curl, and (2) stiff-legged deadlifts.	H/Q functional ratio before and after BEAST90 test	NR	Acknowledges another reliability data/ NR	↔ Functional H/Q ratio before and after BEAST90 post-intervention only in IG1 in dominant leg (*p* = 0.045, n: 0.38) (intervention x match interaction) ↑ Functional H/Q ratio before BEAST90 post-intervention in both IG2 (+14.6%, d = 0.73, *p* = 0.01) and IG1 in dominant leg (+4.9%, d = 0.25, *p* = 0.039)	Supervisor: Experienced S&C coach. IG1 did not change the decline before/after BEAST90, but probably because the increase in the H/Q post- was higher than in IG2.
Rey et al. 2018 [78]	23 male soccer players (age: 24.7 ± 3.8 years) Amateur	**IG:** FIFA 11+ **Duration:** 6 weeks **Frequency:** 3/week **Session duration:** ≈25 min **Training components:** Fifa 11+ consisting on 3 parts: (1) 6 running exercises at low speed; (2) 6 exercises targeting strength, balance, neuromuscular control and core stability with 3 levels of increasing difficulty; (3) running exercises at moderate/high speed.	CG: Standard warm up with jogging, ball exercises and active stretching.	FMS score, divided into FMSmove, FMSflex and FMSstab.	NR	Inter-rater: ICC = 0.899 Intra-rater: ICC = 0.991/ NR	↔ No between groups differences.	Supervisor: Fitness trainer. No *p* values shown.
Riela et al. 2019 [81]	30 male soccer players (IG = 15, age: 23.80 ± 4.6; CG = 15, age: 24.78 ± 2.08 years) Italian 2nd division	**IG:** Movement-based program **Duration:** 8 weeks **Frequency:** 3/week **Session duration:** ≈30 min **Training components:** 15 min of exercises aimed at improving mobility and flexibility and 15 min of stability and posture, and strength with the use of elastic bands, medicine balls and foam rollers.	CG: Standard technical-tactical routing of warm up.	FMS score, divided into advances movement, mobility and stability	NR	NR/ NR	↑ Advanced movement (F(1,28) = 14.43, *p* = 0.03) and mobility (F(1,28) = 3.89, *p* = 0.50) in IG.	Supervisor: Specialised trainer. No *p* values. No counterbalanced the intervention with the two groups.
Whalan et al. 2019 [58]	806 male soccer players (IG1 = 398, age: 24,8; IG = 408, age: 23.8 years) Sub-elite	**IG1:** Rescheduled FIFA 11+ **Duration:** 1 season (28–34 weeks) **Frequency:** 2/week (+ Parts 1 and 3 before matches) **Session duration:** ≈20–25 min **Training components:** 2 parts of the Fifa 11+ performed at the start of the warm-up (parts 1 and 3), and one part performed at the end of training during the cool down period (part 2). In Part 2, players remained at level 1 for a minimum of 2 weeks, and progressed to level 3 after a minimum of 6 weeks	IG2: FIFA 11+ Duration: 1 season (28–34 weeks) Frequency: 2/week (+ Parts 1 and 3 before matches) Session duration: ≈20–25 min Training components: 3 parts of the Fifa 11+ performed at the start of the warm-up. In Part 2, players remained at level 1 for a minimum of 2 weeks, and progressed to level 3 after a minimum of 6 weeks.	Non-contact ACL injury incidence per 1000 h of AE.	IG1 = 18.9 (doses), 32.7% (doses/sessions) IG2 = 29.1 (doses), 57.7% (doses/sessions)	NA	↔ Non-contact ACL injury incidence (*p* = 0.238) in IG2 (IR/1000h = 0.06 [0.01–0.2]) compared to IG1 (IR/1000h = 0.15 [0.01–0.4])	Supervisor not specified. Weeks of season not specified.

ACL—anterior cruciate ligament, FMS—functional movement score, GRF—ground reaction force, AE—athlete exposures, SWC—smallest worthwhile change, IG—intervention group, CG—control group, NR—non-reported, d—Cohens’d, ES—effect size, ESc—effect size calculated through Hedge’s g, CMJ—countermovement jump, DVJ—drop vertical jump, NA—non-applicable, ↓—decrease, ↑—increase, ↔—no change.

**Table 3:** Characteristics of the non-randomised studies included in the systematic review.

Reference	Participants Level	Intervention	Comparison	Outcomes	Compliance Rate	Reliability /SWC	Results	Comments
Malliou et al. 2004 [75]	100 young soccer players (IG = 50, age: 16.7 ± 0.5; CG = 50, age: 16.9 ± 0.7 years) First Greek division	**IG:** Proprioception training program **Duration:** 12 weeks (competition period) **Frequency:** 2/week **Session duration:** ≈20 min **Training components:** Balance exercises (in order to maintain balance while they were performing soccer agilities, such as headers) performed on Biodex Stability System device, mini trampoline and balance boards.	CG: Same training than IG but without any special-extra balance training	Total stability, anterior-posterior and medial-lateral index in a balance test	NR	NR	↓ Total index (ES_c_ = 1.13, *p* < 0.001), A-P Index (ES_c_ = 1.20, *p* < 0.001), and M-L Index (ES_c_ = 0.69, *p* < 0.05) in IG	Supervisor not specified.
Gioftsidou et al. 2008 [67]	68 male soccer players (age: 24.1 ± 5.7 years) Professional	**IG:** Isokinetic training program **Duration:** 8 weeks **Frequency:** 3/week **Session duration:** ≈60 min **Training components:** 10 sets in a velocity spectrum exercise (5 sets with both flexor and extensor muscle groups + 5 sets with only the weak muscle group based on the initial measurement)	CG: NR	Concentric H/Q ratio at 60 and 180 °/s. Differences between limbs in peak torque for flexors and extensors.	NR	NR NR	↑ H/Q ratio at 60 °/s and 180 °/s at both legs (ES_c_ = 0.51–0.87) in IG. ↓ Difference between limbs in peak torque in knee extensors and knee flexors at 60 and 180 °/s (ES_c_ = 1.18–1.75)	Supervisor not specified. Lack of information regarding CG, and weak in the IG. Disparity between groups (IG = 41, CG = 27 players). *p* values and results in CG not specified.
Grooms et al. 2013 [54]	41 male soccer players (CG = 30, age: 20.3 ± 1.6; IG = 34, 20.0 ± 2.4 years) NCAA Division III	**IG:** F-MARC 11+ **Duration:** ≈12 weeks **Frequency:** 5–6/week **Session duration:** ≈20 min **Training components:** (1) running exercises with dynamic stretching and controlled perturbations; (2) strength, balance, and jump-landing control with progression; and (3) higher-speed running drills with cutting manoeuvres.	CG: Standardised warm-up (1st season)	Relative risk (per 1000 athlete-exposures) of ACL injuries	NR	NA	↔ No ACL injury occurred during either season.	Supervisor: Athletic trainer. Low sample to analyse risk ratio of ACL injuries. 7 dropouts in CG
Sliwowski et al. 2015 [79]	24 junior male soccer players (IG = 14, age: 17.0 ± 0.78; CG = 10, age: 17.1 ± 0.71 years) Polish U17 championship	**IG:** RT program specific to imbalance **Duration:** 6 weeks **Frequency:** 2/week **Session duration:** ≈90 min **Training components:** Two parts: (1) a set of 5 reps at 80% of 1RM of 12 upper and lower body exercises, 3–5 min of rest between sets, and (2) 2–3 additional series of 5–7 reps at 80% of 1RM. Depending on the imbalance, the additional exercises were performed for the muscle groups of a given extremity.	CG: Only performed the first part of the RT program	Conventional H/Q ratio for D and ND (54.9% considered deficient) at 60 °/s. Bilateral ratio between knee extensors and flexors at 60 °/s.	NR	NR/ NR	↑ H/Q ratio in D leg (Esc = 0.43, *p* < 0.05) in IG. ↔ No changes in the bilateral differences in any group.	Supervisor: Qualified strength training instructor. Disbalance between groups. Higher H/Q ratio in CG than in IG at baseline.
Ibis et al. 2018 [72]	42 male soccer players (CG = 14, age: 22 ± 1.35; IG1 = 14, age: 23.21 ± 2.29; IG2 = 14, age: 23 ± 1.51 years) Amateur	**IG1:** Strength training group **Duration:** 8 weeks **Frequency:** 3/week **Session duration:** ≈60 min (estimated) **Training components:** 4 series of additional strength training at increasing load (80–95% of 1RM) of 7 lower extremity strength exercises in a pyramidal method. **IG2:** Individual-specific strength training group **Duration:** 8 weeks **Frequency:** 3 weeks **Session duration:** ≈75 min (estimated) **Training components:** Same training than IG1 but with 4 additional series of 2 extra exercises depending on the participant deficiencies (those with H/Q rate low or bilateral flexor deficiency performed two extra knee flexor exercises, and those with bilateral extensor deficit performed two extra knee extensor exercises).	CG: Regular training	Conventional H/Q ratio and bilateral differences (BLD) for hamstrings and quadriceps at 60, 180 and 300°/s	NR	NR/ NR	↓ H/Q ratio at 300 °/s in ND (ES_c_ = 0.58, *p* = 0.026) in CG. ↑ H/Q ratio at 60 °/s in D (ES_c_ = 0.43) in IG1 and in D and ND, 180 °/s in D and ND, 300 °/s in D and ND (ES_c_ = 0.58–1.68) in IG2.	Supervisor not specified. Low sample. 2 training/week CG vs. 5 training/week IG1 and IG2, and also IG2 > IG1 regarding workload.
Arundale et al. 2018 [59]	68 women soccer players (IG = 48, CG = 20 in one testing point; IG = 22, CG = 15 all time points). NCAA Division I and II	**IG:** FIFA 11+ **Duration:** 1 competitive season **Frequency:** 3/week **Session duration:** ≈20 min **Training components:** (1) Running exercises that encompass cutting, COD, decelerating and proper landing techniques; (2) strength, plyometric and balance exercises that focus on core strength, eccentric control and proprioception; (3) running exercises.	CG: Standard warm-up	Peak hip flexion, adduction and internal rotation angles and moments, and peak knee flexion and abduction angles and moments in a DVJ from 40 cm.	NR	ICC = 0.63–0.92/ SDC and MID directly measured from the pre-season data.	1st season: ↔ No differences between groups in postseason peak knee abduction when controlling for baseline differences. No significant time x group interactions for valgus collapse value for either limb. Both groups increased peak knee abduction angle and peak hip abduction an external rotation on the ND (IG) and on the D (CG), but no time x group interaction. ↓ Clinically meaningful decrease (>MID) in peak knee flexion angle in CG for D leg (F(1,35) = 7.64, *p* = 0.05, np2 = 0.18) 2nd season: ↑ ND hip abduction angle (>SDC), ND hip external rotation angle (>SDC), D knee abduction angle (>MID) in 1st season vs. 2nd season, and ND peak hip flexion angle (>SDC) in 2nd season vs. 1st season (time x season). ↓ ND hip flexion angle (>SDC) in 1st vs. 2nd season (time x season). ↔ No significant time x season interaction in valgus collapse in either leg.	Supervisor: Athletic trainer.
Dos’Santos et al. 2019 [66]	26 male soccer players (IG = 13, age: 16.9 ± 0.2; CG = 13, age: 17.8 ± 0.3) Professional club (U17)	**IG:** COD speed and technique modification **Duration:** 6 weeks **Frequency:** 2/week **Session duration:** 20 min **Training components:** COD velocity and technique modification	CG: Regular field-based warm-up	CMAS score in a 90° COD with both limbs	IG = 88.5% CG = 90%	ICC = 0.774–0.934 SEM = 0.49–0.69 CV = 11.4–22.2/ SDD = 1.36–1.84	↓ CMAS in right (*p* = 0.025, g = −0.85, −22.5%), and left (*p* = 0.018, g = −1.46, −33.9%) legs in IG. ↔ No change in CG.	Supervisor: Certified S&C specialist. Some dropouts of participants in the IG (*n* = 5).
Krutsch et al. 2020 [55]	1527 male football players (IG = 529, age: 22.7 ± 4.3, CG = 601, age: 21.9 ± 4.1 years) Elite	**IG:** Newly implemented football-specific training modules **Duration:** one season **Frequency:** At least 2/week **Session duration:** ≈12 min **Training components:** One main exercise and several alternative with specific variations in movements and techniques (established by the researchers; decided by the coach) of 5 modules: mobilisation, core stability, leg axis stability, jumping, and landing exercises and agility.	CG: Their usual training program	Severe knee and ACL/PCL injury incidence per 1000 AE	NR	NA	↓ Severe knee injury incidence (0.38 vs. 0.69/1000h, *p* < 0.05) in IG compared to CG. ↔ ACL/PCL injury incidence (0.11 vs. 0.18/1000h, *p* > 0.05) in IG compared to CG	Supervisor: Coach. High number of dropouts, but do not specify how many of each group. Weeks of intervention not specified.

ACL—anterior cruciate ligament, PCL—posterior cruciate ligament, AE—athlete exposures, CMAS—cutting movement assessment score, D—dominant, ND—nondominant, SDC—smallest detectable change, COD—change of direction, SWC—smallest worthwhile change, ICC—intraclass correlation coefficient, CV—coefficient of variation, IG—intervention group, CG—control group, NR—non-reported, ES—effect size, ESc—effect size calculated through Hedge’s g, NA—non-applicable, ↓—decrease, ↑—increase, ↔—no change.

**Table 4:** Characteristics of the single-arm studies included in the systematic review.

Reference	Participants Level	Intervention	Outcomes	Compliance Rate	Reliability /SWC	Results	Comments
Holcomb et al. 2007 [71]	12 female soccer players (age: 20 ± 0.8 years) NCAA 1st division	**IG:** Hamstring-emphasised RT **Duration:** 6 weeks **Frequency:** 2/week **Session duration:** Unable to determine **Training components:** Two of different exercises (single leg curls, straight leg dead lifts, good morning exercises, trunk hyperextensions, resisted sled walking and exercise ball leg curls) in addition to speed, agility and endurance training.	Functional and conventional H/Q ratio at 240, 180 and 60 °/s	NR	NR NR	↑ Functional H/Q (ES_c_ = 1.13, *p* = 0.049). ↔ Conventional H/Q (ES_c_ = 1.26, *p* = 0.172)	Supervisor not specified. Low sample. Results not stratified by velocities. Poor description of the intervention.
Brito et al. 2010 [61]	20 male soccer players (age: 22.3 ± 4,2 years) Sub-elite	**IG:** FIFA 11+ **Duration:** 10 weeks **Frequency:** 3/week **Session duration:** 20 min **Training components:** (1) Running, stretching and controlled contacts; (2) strengthening, plyometrics and balance; and (3) speed running and soccer-specific movements.	H/Qcon 60° and 180°, H/Qecc30° and Hecc30 °/Qcon180°(DCR) ratios in isokinetic tests in both limbs	73%	Acknowledges another reliability data/ NR	↑ H/Qcon60° (ES_c_ = 0.11) and Hecc30°/Qcon180° (ES_c_ = 0.39) in non-dominant limb (*p* < 0.05).	Supervisor not specified. 2 dropouts.
McCann et al. 2011 [76]	10 healthy female soccer players (age: 19.1 ± 0.9 years)	**IG:** Resistance and conditioning training **Duration:** 10 weeks (11 weeks for retention) **Frequency:** 4/week **Session duration:** ≈60 min **Training components:** Strength, endurance or RT twice a week (depending on the athletes’ weaknesses) and conditioning training (speed, quickness, plyometric and agility drills) twice a week.	Knee abduction and hip abduction angles, and knee flexion moment in a running stop jump.	NR	NR/ NR	↑ Hip abduction angle and knee flexion moment from pre- to post-intervention. ↓ Knee abduction angle from pre- to post-intervention and retention (Z = −2.29), and hip abduction angle and knee flexion moment from post-intervention to retention.	Supervisor: CSCS specialist. No descriptive data. Low cohort (*n* = 10). No information about training during retention.
Greska et al. 2012 [70]	12 female soccer players (age: 19.2 ± 0.8 years) NCAA 1st division	**IG1:** Strength focused RT **Duration:** 10 weeks **Frequency:** 4/week **Session duration:** ≈60 min **Training components:** 2 days of low volume of RT exercises with a self-selected rest interval and with augmented feedback (verbal and visual) in relation to the movement patterns and body positioning. 2 days of field conditioning focusing on speed, quickness, plyometric and agility drills. **IG2:** Endurance focused RT **Duration:** 10 weeks **Frequency:** 4/week **Session duration:** ≈60 min **Training components:** 2 days of high volume of RT exercises with a 30-s rest interval between each exercise and with augmented feedback (verbal and visual) in relation to the movement patterns and body positioning. 2 days of field conditioning focusing on speed, quickness, plyometric and agility drills. **IG3:** Maintenance focused RT **Duration:** 10 weeks **Frequency:** 4/week **Session duration:** ≈60 min **Training components:** 2 days of hybrid scheme between IG1 and IG2, performing strength-focused 1 day and endurance-focused the other day. 2 days of field conditioning focusing on speed, quickness, plyometric and agility drills.	Kinetic and kinematic variables during a stop-jump task	95%	NR/ NR	↓ Knee abduction angle at IC (d = 0.76, *p* = 0.007) ↑ Hip abduction angle at IC (d = 0.63, *p* = 0.007) and peak knee flexion (d = 0.99, *p* = 0.002) and maximum knee extension moment (ES_c_ = 0.59, *p* = 0.022) at peak stance.	Supervisor: CSCS specialist. All participants measured together (not by group). Low sample. Inadequate and unbalanced group sizes. Differences in BW at baseline. Too much time required.
Lehnert et al. 2017 [74]	18 male soccer players (age: 17.1 ± 0.4 years) Czech 1st division	**IG:** Pre-season training with the inclusion of progressive eccentric hamstring exercises **Duration:** 10 weeks **Frequency:** from 1/week (from 1st to 4th) to 3/week (from 5th to 10th) **Session duration:** ≈5–15 min **Training components:** Strength training with a special focus on eccentric hamstring exercises such as the Nordic curl (from 1 set of 5 reps at the beginning of the program, to 3 sets of 8–12 repetitions by the 5th week)	Conventional and functional H/Q ratio at 60 °/s.	NR	NR/ NR	↑ Functional H/Q in ND (ES_c_ = 0.62, *p* < 0.05). ↔ Conventional H/Q in D and ND (*p* > 0.05)	Supervisor not specified.
Oshima et al. 2018 [77]	8 male soccer players (age: 20.4 ± 0.5 years) Collegiate	**IG:** FIFA 11+ (part 2) **Duration:** 24 weeks (6 months) **Frequency:** ≥3/week **Session duration:** ≈10 min **Training components:** Three levels of difficulty of six exercises aiming to increase muscular strength (core and lower limbs), balance, muscle control (plyometrics), and core stability.	Postural sway for 60s: Length per time (LG) and environmental area (AR) (two-leg stance with eyes opened and then with eyes closed and single leg standing with eyes opened). Star excursion balance test (SEBT - 8 directions). H/Q ratio	NR	NR/ NR	↑ Anterior-lateral with D and medial, posterior-medial, and posterior with ND (ES_c_ = 0.38–0.71) in SEBT. ↔ H/Q ratio in D (ES_c_ = 0.20, *p* > 0.05) and ND (ES_c_ = 0.44, *p* > 0.05)	Supervisor not specified. No *p* values reported. Low sample (*n* = 8)

CG—control group, ES—effect size, ESc—effect size calculated through Hedge’s g, IG—intervention group, NA—non-applicable, NR—non-reported, RT—resistance training, SWC—smallest worthwhile change, D—dominant, ND—nondominant, ↓—decrease, ↑—increase, ↔—no change.

**Table 5:** Summary of evidence regarding the efficacy of exercise-based interventions on ACL injury incidence and risk factors of ACL injury coming from randomized-controlled trials and nonrandomized studies.

Injury Incidence	Risk Factors of ACL Injury
FIFA 11+ [56,57] and PEP Program [53] appears to be effective at reducing ACL injury incidence. FIFA 11+ still present some pitfalls that restricts its implementation in football teams due to duration, boredom and soreness issues [85,86]. The programme proposed by Krutsch et al. [55] appears to be an effective and feasible option to decrease severe knee injuries, but should be further investigated through a RCT specifically investigating ACL incidence. The high sample size or time needed to permit a high ACL injury occurrence which satisfies statistical power is extremely difficult to achieve [53], and thus, complicating the development of rigorous intervention studies investigating their effect in ACL injury incidence.	FIFA 11+ may be effective by eliciting positive adaptations in terms of balance [60,65] or dynamic stabilization [73], but not in mobility, asymmetry in hop tests [60], fundamental movement patterns [78] or biomechanics of jump landing tasks [59] in adult football players. The core stability programme proposed by Dello Iacono et al. [65] seems to be effective at either improving H/Q ratio and GRF asymmetry during CMJ. The balance programme proposed by Gioftsidou et al. [68] may be effective at eliciting moderate to large improvements in balance. The TM program proposed by Dos’Santos et al [66] appears to be effective at improving both performance and movement quality of cutting actions (main mechanism of ACL injuries [18]). All of the above interventions appear to provide the best effectiveness/feasibility balance to be implemented in the real soccer context, although they should be further explored through low risk of bias RCT designs.

ACL anterior cruciate ligament, CMJ countermovement jump, GRF ground reaction force, H/Q hamstrings to quadriceps, RCT randomized controlled trial, TM technique modification.

**Table 6:** Summary of considerations and recommendations for future research.

Recommendation	Rationale
**Developing preventative frameworks focusing on movement quality in risky movements**	Of the 29 studies included, only four evaluated the effect of any exercise-based intervention on movement quality during potentially risky movements associated with the common ACL injury mechanism in football [59,66,70,76], while only one study has been carried out with a cost-effective tool which could be easily implemented in the football context [66]. Even though the effect of several interventions on movement quality has been performed with different team sports including football players [37], the specific nature of some exercise-based adaptations and the uniquely context in which football occur [119] could justify the need of developing more football-specific preventative programs. Furthermore, only one NRCT study incorporated TM as a strategy to mitigate risk factors of ACL injury [66] despite it is widely known the contribution of biomechanical determinants in increasing ACL loads [120], and knowing its promising results in other sports [121] and in young female players [122]. On the other hand, given the influence that neurocognition may have in ACL injuries [123], open skills tasks that evaluate athletes under football-specific neurocognitive demands (e.g., unanticipated COD) should be included in screening tests assessing effectiveness of intervention programs [123].
**Improving quality of the interventions**	None of the included studies had pre-registered the study protocol before to its execution, by which it may be speculated that this is not common trend in Sports Sciences. Since this pre-registration would allow to compare evaluation and data analysis finally carried out with those initially intended, it would be easier to detect risk of bias, especially that related to bias in the se-lection of reported outcomes [49]. Although problems arise from the inability of blinding athletes and care providers are sometimes unavoidable in the context of interventions carried out in football teams, others such us bias due to confounding variables in NRCT or bias due to deviations from intended interventions and unequal training volumes in RCT can be prevented with appropriate analysis performed (i.e., pre-registration and overall transparency with the research process) [50]. Randomization and concealment of allocation sequence processes should be improved and explicitly reported. By doing this, the number of low risks of bias studies (only one in the present review) would be higher and, therefore, findings more reliable [49]. Additionally, by increasing samples and/or the follow-up times, greater statistical power would be reached in the associations, especially those with ACL injury incidence [21].
**Appropriate reporting of important features of the program: reliability of outcomes, SWC, compliance and supervisor**	It is also suggested that reliability and SWC data are directly measured so that practical relevance of the results obtained because of an exercise-based intervention could be determined [90], and to ensure that training induced changes exceed the measurement error to increase the certainty improvements are “real”. This is extremely pertinent when researchers do not have the opportunity to utilize a CG and therefore adopt a single-arm design. In the present review, only 5/24 and 3/24 of the studies evaluating risk of injury variables have reported directly measured reliability and SWC data, respectively. Of note, it is suggested that supervisor of the interventions is specified, as it is known the potential positive influence of the quality of the feedback provided (i.e., through verbal, auditory and visual cues) [124,125,126] in the reinforcement of proper technique during anterior cruciate ligament injury prevention exercises [127]. 17/29 included studies reported who were the intervention supervisors. Additionally, despite the positive relationship that has been shown between compliance and effectiveness of exercise-based interventions targeting injury reduction [57,58], only 8/29 studies reported compliance rates. Indeed, compliance to the intervention has been shown to be a critical component of prevention programmes, as it highly determines its effectiveness [87]. Therefore, going forward, before concluding a training modality as potentially ineffective, it is central to consider the training compliance which, unfortunately, 72% of studies in this review failed to report. Thus, it is suggested to incorporating such data in future research to confirm the efficacy of injury mitigation training interventions.

ACL anterior cruciate ligament, CG control group, NRCT nonrandomized studies, RCT randomized controlled trial, SWC smallest worthwhile change.

## Data Availability

The data within this systematic review and is available through the relevant articles referenced throughout.

## Supplementary Materials

The following are available online at www.mdpi.com/xxx/. Risk of bias in individual studies, Table S1: Risk of bias in different domains of randomised controlled trials (i.e., RoB 2), Table S2: Risk of bias in different domains of non-randomised trials (i.e., ROBINS-I), Figure S1: Risk of bias graph of the randomised-controlled trials (RCT) (i.e., Cochrane’s RoB 2), Figure S2: Risk of bias graph of the nonrandomised studies (NRCT) (i.e., Cochrane’s ROBINS-I).
