# Supplementary material for: Exercise-Based Training Strategies to Reduce the Incidence or Mitigate the Risk Factors of Anterior Cruciate Ligament Injury in Adult Football (Soccer) Players: A Systematic Review
Source: Int J Environ Res Public Health. 2021 Dec 18;18(24):13351. doi: 10.3390/ijerph182413351 (PMC8704173; doi:10.3390/ijerph182413351)
Supplement: Supplementary file 1 [file ijerph-18-13351-s001.zip › ijerph-1459938-supplementary.pdf]

Article name: Exercise-based training strategies to reduce the incidence or mitigate the risk factors of anterior cruciate ligament injury in adult football (soccer) players: a systematic review.

Journal name: International Journal of Environmental Research and Public Health.

Authors: Jesús Olivares-Jabalera[1,2*], Alberto Fílter-Ruger[2], Thomas Dos´Santos[2,3], Jose Afonso[4], Francesco Della Villa[5], Jaime Morente-Sánchez[2], Víctor Manuel Soto Hermoso[1], Bernardo Requena[1,2]

1 HUMAN Lab, Sport and Health University Research Institute (iMUDS), University of Granada, Granada, Spain
2 FSI Sport Research Lab, Granada, Spain
3 Department of Sport and Exercise Sciences, Musculoskeletal Science and Sports Medicine Research Centre, Manchester Metropolitan University, All Saints Building, Manchester Campus John Dalton Building, Manchester Campus,
Manchester M15 6BH, UK
4 Centre for Research, Education, Innovation and Intervention in Sport, Faculty of Sports of the University of Porto, Rua Dr. Plácido Costa, 91, 4200-450 Porto, Portugal
5 Education and Research Department, Isokinetic Medical Group, FIFA Medical Centre of Excellence, Bologna, Italy

*** Corresponding author information:**
C/Menéndez Pelayo, 32, 18016, Granada, Spain (iMUDS, University of Granada)
jesusyolivares@gmail.com
+34 626 02 45 33

Appendix 1 Risk of Bias in individual studies

A graph showing the risk of bias in both parallel and cluster RCT at the different domains level of the RoB 2 tool is displayed in Fig. A1. Only one study is reported to be at low overall risk of bias (1), while 4 present some concerns (2–5) and 10 are at high risk of bias (6–15). For a study to be considered at low overall risk of bias in RCT, it is required that all its individual domains present low risk of bias. In the included studies, none of them had pre-registered the study protocol. Given that with no protocol available it is not possible to compare the analyses performed to those initially intended, the 15 RCT were automatically judged at some concerns in the domain "bias in selection of the reported result". Despite this, the study carried out by Impellizzeri (1) was considered to be at low risk of bias. Given that none of the studies had pre-registered the study protocol, and this was the only study with low risk of bias in all the other domains, a manual override of the algorithm (MOR) was conducted, a possibility that is predicted in Cochrane's guidelines (16). The domain at the lowest risk of bias of the different studies was "bias in measurement of the outcome", in which 12/15 studies achieved a low risk of bias given that, generally, outcome measurement methods were appropriate and testers blinders or measurements objective. On the contrary, the domain at the highest risk of bias was "bias due to deviations from intended interventions", in which 8/15 studies were reported at high risk of bias. This domain assesses if there are deviations from the intended interventions, likely affecting the outcome, and if there are inappropriate (i.e., per-protocol) or no information regarding the analysis used to estimate the effect of assignment to interventions, issues which placed some studies of the systematic review to high risk of bias (6–9,11,12,14,15). Given that intended-to-treat are preferred over per-protocol analyses when aiming at evaluating the effectiveness of an intervention in the real context (i.e., football teams), per-protocol analyses were considered inappropriate for the purpose of the present review (17). An additional domain was used for assessing the risk of bias of cluster RCT (i.e., risk of bias arising from the timing of identification or recruitment of participants), being in all of them judged as low risk of bias (6,9,12,14).

Regarding the 8 non-RCT, 6 of them were judged as critical risk of bias (18–23), while two were judged at moderate risk of bias (24,25) (Fig. A2). The most problematic domain is the risk of bias due to confounding, where the 6 studies at critical overall risk of bias also present critical (19–23) or serious (18) risk of bias due to poor or no control of confounders (i.e., relevant prognostic variables that could have predicted intervention received at baseline). Those studies should be considered with caution. On the other hand, the domains "bias in classification of interventions" and "bias in measurement of outcomes" were the least problematic, with all studies being judged at low risk of bias. It must be noted that in DosSantos et al. (25) study, stricty following the ROBINS-I tool, the algorithm would require a global assessment of serios risk since one dimension is at serios risk. However, in the other six dimensions, this study presents one "moderate risk" and five "low risk". Also, in the category that had serious risk, it is possible that true value did not interfere. Additionally, having some number of dropouts in the context of football teams is quite common. Therefore, these are the reasons why authors have finally considered judging it at "moderate" overall risk of bias.

3.3.1 Single-arm studies

Although the aim of the review was to provide evidence coming from all study designs, single arm studies do not provide reliable measures of the effects of an intervention. In fact, the effects may have been: (i) produced or helped by the intervention; (ii) independent of the intervention; or (iii) could have been better without the intervention (26). Therefore, single arm studies should be used to assess feasibility and safety of interventions, while establishing some of the problems associated with their implementations. However, the results should be interpreted with caution. Indeed, the results from single arm studies should not be used to assess the efficacy of the interventions (27). Even if randomization is not possible, multi-arm studies will at least provide a greater degree of understanding, as comparisons will be possible. In this context, single-arm studies, if used for purposes other than assessing feasibility and safety, should be considered at critical risk of bias (26). Therefore, single-arm studies will be only discussed as potentially feasible implementations whose effectiveness should be further investigated.

Table S1. Risk of bias in different domains of randomized controlled trials (i.e. RoB 2).

Study	Bias arising from the randomization process	Bias arising from the timing of identification or recruitment of participants	Bias due to deviations from intended interventions (ITT)	Bias due to missing outcome	Bias in measurement of the outcome	Bias in selection of the reported result	Overall
Gilchrist et al. (2008)	!	+	-	-	+	!	-
Steffen et al. (2008)	!	-	+	+	+	!	!
Brughelli et al. (2010)	+	-	!	!	+	!	!
Daneshjoo et al. (2012)	-	-	-	-	-	!	-
Gioftsidou et al. (2012)	!	-	-	+	+	!	-
Impellizzeri et al. (2013)	+	-	+	+	+	!	+ *MOR
Silvers et al. (2015)	+	+	-	-	+	!	-
Dello Iacono et al. (2016)	!	-	+	+	+	!	!
Gonzalez-Jurado et al. (2016)	!	-	+	+	-	!	-
Ayala et al. (2017)	-	-	-	-	+	!	-
Silvers et al. (2017)	+	+	-	-	+	!	-
Delextrat et al. (2018)	-	-	+	-	+	!	-
Rey et al. (2018)	+	-	+	+	+	!	!
Whalan et al. (2019)	!	+	-	+	+	!	-
Riela et al. (2019)	!	-	-	+	-	!	-

\+ = low risk of bias; ! = some concerns; - = high risk of bias; *ITT* intention-to-treat; *MOR* manual override of the algorithm

Table S2. Risk of bias in different domains of non-randomized trials (i.e. ROBINS-I).

Study	Pre-intervention: bias due to confounding	Pre-intervention: bias in selection of participants into the study	At intervention: bias in classification of interventions	Post intervention: bias due to deviations from intended interventions (ITT)	Post-intervention: bias due to missing data	Post-intervention: bias in measurement of outcomes	Post-intervention: bias in selection of the reported result	Overall
Malliou et al. (2004)	+	+	+	+	NI	+	!	!
Gioftsidou et al. (2008)	- -	-	+	+	+	+	!	- -
Grooms et al. (2013)	- -	-	+	+	+	+	!	- -
Sliwowski et al. (2015)	- -	+	+	+	NI	+	!	- -
Ibis et al. (2018)	- -	+	+	- -	NI	+	!	- -
Arundale et al. (2018)	- -	!	+	- -	- -	+	!	- -
Dos'Santos et al. (2019)	+	+	+	+	-	+	!	! *MOR
Krutsch et al. (2020)	-	+	+	- -	- -	+	!	- -

\+ = low risk of bias; ! = moderate risk of bias; - = serious risk of bias; - - = critical risk of bias. *ITT* intention-to-treat, *MOR* manual override of the algorithm, *NI* no information.

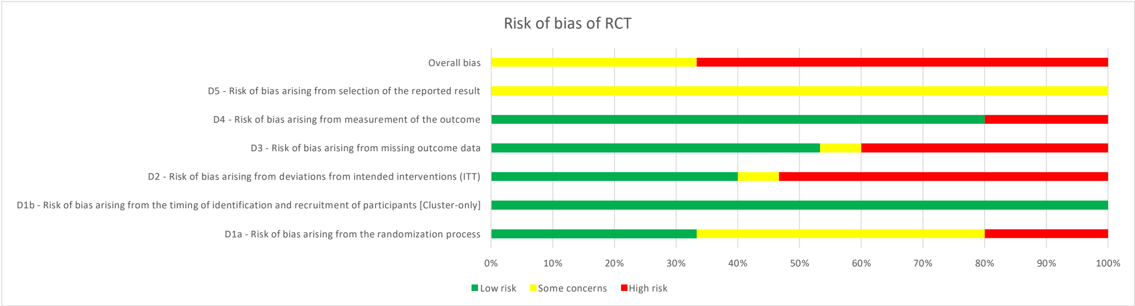

Supplementary figure S1. Risk of bias graph of the randomized-controlled trials (RCT) (i.e. Cochrane's RoB 2).

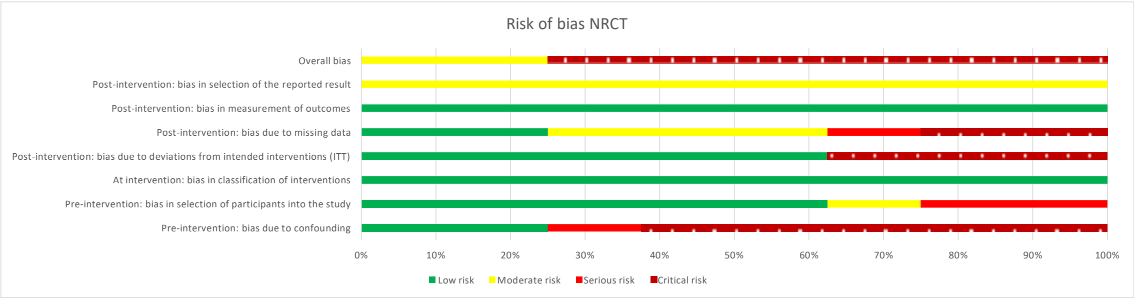

Supplementary figure S2. Risk of bias graph of the nonrandomized studies (NRCT) (i.e. Cochrane's ROBINS-I).

References

1. Impellizzeri FM, Bizzini M, Dvorak J, Pellegrini B, Schena F, Junge A. Physiological and performance responses to the FIFA 11+ (part 2): A randomised controlled trial on the training effects. J Sports Sci. 2013;31(13):1491–502.
2. Steffen K, Bakka HM, Myklebust G, Bahr R. Performance aspects of an injury prevention program: A ten-week intervention in adolescent female football players. Scand J Med Sci Sport. 2008;18(5):596–604.
3. Brughelli M, Mendiguchia J, Nosaka K, Idoate F, Arcos AL, Cronin J. Effects of eccentric exercise on optimum length of the knee flexors and extensors during the preseason in professional soccer players. Phys Ther Sport [Internet]. 2010;11(2):50–5. Available from: http://dx.doi.org/10.1016/j.ptsp.2009.12.002
4. Dello Iacono A, Padulo J, Ayalon M. Core stability training on lower limb balance strength. J Sports Sci [Internet]. 2016;34(7):671–8. Available from: http://dx.doi.org/10.1080/02640414.2015.1068437
5. Rey E, Padrón-Cabo A, Penedo-Jamardo E, González-Víllora S. Effect of the 11+ injury prevention programme on fundamental movement patterns in soccer players. Biol Sport. 2018;35(3):229–36.
6. Gilchrist J, Mandelbaum BR, Melancon H, Ryan GW, Silvers HJ, Griffin LY, et al. A randomized controlled trial to prevent noncontact anterior cruciate ligament injury in female collegiate soccer players. Am J Sports Med. 2008;36(8):1476–83.
7. Daneshjoo A, Mokhtar AH, Rahnama N, Yusof A. The Effects of Comprehensive Warm-Up Programs on Proprioception, Static and Dynamic Balance on Male Soccer Players. PLoS One. 2012;7(12):1–10.
8. Gioftsidou A, Malliou P, Pafis G, Beneka A, Tsapralis K, Sofokleous P, et al. Balance training programs for soccer injuries prevention. J Hum Sport Exerc. 2012;7(3):639–47.
9. Silvers-Granelli H, Mandelbaum B, Adeniji O, Insler S, Bizzini M, Pohlig R, et al. Efficacy of the FIFA 11+ injury prevention program in the collegiate male soccer player. Am J Sports Med [Internet]. 2015;43(11):2628–37. Available from: https://doi.org/10.1177/0363546515602009
10. Gonzalez-Jurado JA, Romero Boza S, Campos Vázquez MA, Toscano Bendala FJ, Otero-Saborido FM. Comparación de un entrenamiento propioceptivo sobre base estable y base inestable / Comparison of a Proprioceptive Training Program on Stable Base and Unstable Base. Rev Int Med y Ciencias la Act Física y del Deport. 2016;64(2016):617–32.
11. Ayala F, Pomares-Noguera C, Robles-Palazón FJ, Del Pilar García-Vaquero M, Ruiz-Pérez I, Hernández-Sánchez S, et al. Training Effects of the FIFA 11+ and Harmoknee on Several Neuromuscular Parameters of Physical Performance Measures. Int J Sports Med. 2017;38(4):278–89.
12. Silvers-Granelli HJ, Bizzini M, Arundale A, Mandelbaum BR, Snyder-Mackler L. Does the FIFA 11+ Injury Prevention Program Reduce the Incidence of ACL Injury in Male Soccer Players? Clin Orthop Relat Res. 2017;475(10):2447–55.
13. Delextrat A, Piquet J, Matthews MJ, Cohen DD. Strength-endurance training reduces the hamstrings strength decline following simulated football competition in female players. Front Physiol. 2018;9(AUG):1–12.
14. Whalan M, Lovell R, Steele JR, Sampson JA. Rescheduling Part 2 of the 11+ reduces injury burden and increases compliance in semi-professional football. Scand J Med Sci Sport. 2019;29(12):1941–51.
15. Riela LA, Bertollo M. The effectiveness of eight weeks of a movement-based program on functional movement patterns in male professional soccer players. J Phys Educ Sport. 2019;19(5):1976–83.
16. Higgins JPT, Altman DG, Gøtzsche PC, Jüni P, Moher D, Oxman AD, et al. The Cochrane

Collaboration's tool for assessing risk of bias in randomised trials. BMJ. 2011;343(7829):1–9.
17. Sterne JAC, Savović J, Page MJ, Elbers RG, Blencowe NS, Boutron I, et al. RoB 2: A revised tool for assessing risk of bias in randomised trials. BMJ. 2019;366:1–8.
18. Krutsch W, Lehmann J, Jansen P, Angele P, Fellner B, Achenbach L, et al. Prevention of severe knee injuries in men's elite football by implementing specific training modules. Knee Surgery, Sport Traumatol Arthrosc [Internet]. 2020;28(2):519–27. Available from: https://doi.org/10.1007/s00167-019-05706-w
19. Grooms DR, Palmer T, Onate JA, Myer GD, Grindstaff T. Soccer-specific warm-up and lower extremity injury rates in collegiate male soccer players. J Athl Train. 2013;48(6):782–9.
20. Sliwowski R, Jadczak Ł, Hejna R, Wieczorek A. The Effects of Individualized Resistance Strength Programs on Knee Muscular Imbalances in Junior Elite Soccer Players. PLoS One. 2015;10(12):1–14.
21. Ibis S, Aktuğ ZB, Iri R. Does individual-specific strength training have an effect upon knee muscle strength balances? Knee muscle strength balances. J Musculoskelet Neuronal Interact. 2018;18(2):183–90.
22. Arundale AJH, Silvers-Granelli HJ, Marmon A, Zarzycki R, Dix C, Snyder-Mackler L. Changes in biomechanical knee injury risk factors across two collegiate soccer seasons using the 11+ prevention program. Scand J Med Sci Sport. 2018;28(12):2592–603.
23. Gioftsidou A, Ispirlidis I, Pafis G, Malliou P, Bikos C, Godolias G. Isokinetic strength training program for muscular imbalances in professional soccer players. Sport Sci Health. 2008;2(3):101–5.
24. Malliou P, Gioftsidou A, Pafis G, Beneka A, Godolias G. Proprioceptive training (balance exercises) reduces lower extremity injuries in young soccer players. J Back Musculoskelet Rehabil. 2004;17(3–4):101–4.
25. Dos'Santos T, McBurnie A, Comfort P, Jones PA. The Effects of Six-Weeks Change of Direction Speed and Technique Modification Training on Cutting Performance and Movement Quality in Male Youth Soccer Players. Sports. 2019;7(9):205.
26. Cucherat M, Laporte S, Delaitre O, Behier J-M, d'Andon A, Binlich F, et al. From single-arm studies to externally controlled studies. Methodological considerations and guidelines. Therapies [Internet]. 2020;75(1):21–7. Available from: https://doi.org/10.1016/j.therap.2019.11.007
27. Evans SR. Clinical trial structures. J Exp Stroke Transl Med. 2010;3(1):8–18.
